# 

**Published:** 1868-05

**Authors:** 


MAY, 1868.

### THE AMERICAN JOURNAL OF DENTAL SCIENCE,

EDITED BY

A. SNOWDEN PIGGOT, M.D.,

AND

F. J. S. GORGAS, M.D., D.D.S.

VOL. 2.—THIRD SERIES.—NO. 1.

BALTIMORE:

SNOWDEN & COWMAN.

LONDON:

TRUBNER & CO., 12 PATERNOSTER ROW.

1867.

$3.00 PER ANNUM, PAYABLE IN ADVANCE.

PUBLISHED MONTHLY.

SENT POSTAGE FREE, WHEN PAID FOR IN ADVANCE.

